# Fluorescence-guided versus non-fluorescence-guided resection in high-grade glioma: a systematic review and meta-analysis of survival outcomes

**DOI:** 10.1007/s10143-026-04297-8

**Published:** 2026-04-23

**Authors:** Sai Sanikommu, Alejandro N. Santos, Bashar Dawoud, Ricardo J. Komotar, Victor M. Lu

**Affiliations:** Department of Neurosurgery, Miller School of Medicine, University of Miami, 1501 NW 10Th Avenue, 832 G, Miami, FL 33136 USA

**Keywords:** Fluorescence-guided surgery, High-grade glioma, Glioblastoma, 5-aminolevulinic acid (5-ALA), Overall survival, Progression-free survival, Meta-analysis

## Abstract

Fluorescence-guided resection (FGR) in high-grade glioma surgeries improves tumor visualization and resection extent. Although resection completeness is well documented, its impact on survival varies across studies due to confounding factors and heterogeneity. We conducted a systematic review and meta-analysis to clarify the relationship between fluorescence guidance and survival, distinguishing unadjusted from adjusted effects. A systematic search of PubMed, Embase, Scopus, and Web of Science found studies comparing FGR and non-FGR in adult high-grade glioma patients. Overall survival (OS) and progression-free survival (PFS) were analyzed separately. Risk of Bias was assessed with ROBINS-I and RoB 2, and evidence certainty with GRADE. Seventeen studies involving a total of 2,937 patients (1,466 who underwent FGR and 1,471 who underwent non-FGR) met the inclusion criteria. In unadjusted analyses, FGR was associated with a statistically significant improvement in overall survival (HR 0.72, 95% CI [0.57–0.91]; *I*^*2*^ 47.2%), compared to non-FGR. However, in analyses adjusted for key prognostic variables, this association was diminished and no longer reached statistical significance (aHR 0.63, 95% CI [0.30–1.30]; *I*^*2*^ 80.3%). Progression-free survival analyses indicated trends toward benefit, although these were not statistically significant in both unadjusted (HR 0.70, 95% CI [0.26–1.89]; *I*^*2*^ = 57.9%) and adjusted models (aHR 0.86, 95% CI [0.29–2.54]; *I*^*2*^ = 80.3%), with notable heterogeneity observed. Subgroup analyses comparing 5-ALA and white-light surgery yielded similar patterns, with effects attenuated following adjustment. The certainty of evidence ranged from low to very low. FGR initially demonstrated superior survival outcomes in high-grade glioma; however, the significance diminished upon adjustment for confounders. It primarily serves as an adjunct to surgical procedures to facilitate safe and maximal resection rather than directly enhancing survival. Further randomized investigations incorporating molecular stratification are essential to elucidate its influence on long-term survival.

**Supplementary Information:**

The online version contains supplementary material available at 10.1007/s10143-026-04297-8.

## Introduction

Fluorescence-guided resection (FGR) has become an increasingly utilized intraoperative adjunct in the surgical management of high-grade gliomas, with the aim of enhancing visualization of tumor margins and enabling maximal safe resection [1, 2]. Agents such as 5-aminolevulinic acid (5-ALA) and fluorescein sodium facilitate real-time differentiation between tumor and normal brain tissue, surpassing the capabilities of conventional white-light microscopy [1, 3–5]. Multiple randomized [1, 6, 7] and observational studies [8–17] have demonstrated that fluorescence guidance increases the rate of gross total resection and reduces residual enhancing tumor volume. These technical endpoints are strongly associated with oncologic control and have contributed to the widespread adoption of fluorescence-guided techniques in contemporary neurosurgical practice [1, 18–20].

Despite its increasing application, the effect of FGR on survival outcomes remains inconsistent across various studies [1, 6–17]. While several investigations have reported improvements in overall survival (OS) [8, 9, 12] and progression-free survival (PFS) [1, 11, 13] among patients undergoing fluorescence-guided surgery, others have observed attenuated or absent survival differences when compared with non-fluorescence-guided resection [6, 7, 10, 14–17]. These discrepancies likely reflect significant heterogeneity in study design, patient selection, fluorescence modality, and adjustment for prognostic factors such as performance status, tumor location and volume, molecular characteristics, extent of resection, adjuvant therapy, and treatment era [4, 20, 21]. Significantly, fluorescence-guided surgery is frequently utilized in patients exhibiting more favorable baseline characteristics or within specialized centers, thus raising concerns that the observed survival benefits may be confounded by indication rather than reflecting a direct, independent effect of fluorescence guidance [4, 22].

Given these uncertainties, the degree to which FGR provides an independent survival benefit remains ambiguous. Previous systematic reviews and meta-analyses have primarily aggregated studies with heterogeneous designs and have largely depended on unadjusted survival estimates, thereby constraining the capacity to establish causal relationships [4, 23, 24]. To address this gap, we conducted a systematic review and meta-analysis comparing fluorescence-guided versus non-FGR in high-grade glioma, explicitly separating unadjusted and adjusted hazard ratios for OS and PFS and performing prespecified subgroup analyses of 5-ALA versus white-light surgery. By distinguishing unadjusted associations from adjusted effects, this study aims to clarify whether fluorescence guidance independently influences survival or primarily serves as a surgical adjunct that enhances resection and local disease control.

## Methods

This systematic review and meta-analysis followed the PRISMA 2020 guidelines. The protocol was registered prospectively in the PROSPERO international database of systematic reviews (registration number: 1282250). The study aimed to compare survival outcomes between FGR and non-FGR in patients with high-grade glioma, with separate synthesis of unadjusted and adjusted time-to-event estimates.

Data from this study is available upon reasonable request from the corresponding author.

### Search strategy and eligibility criteria

We conducted a comprehensive literature review across PubMed (MEDLINE), Embase, and Scopus from their inception until January 2026 to identify studies assessing the prognostic significance of FGR in comparison to non-FGR for high-grade glioma brain tumors. The complete search strategy is reported in Supplementary Table S1. Eligible studies met the following criteria: (1) enrolled adult patients (≥ 18 years) with high-grade glioma (WHO grade III–IV or glioblastoma); (2) compared fluorescence-guided resection (using 5-ALA, fluorescein sodium, or mixed fluorescence modalities) with non-fluorescence-guided resection (white-light microscopy or other non-fluorescence comparators); (3) reported OS and/or PFS outcomes; and (4) provided effect estimates as hazard ratios (HRs) with corresponding 95% confidence intervals (CIs), or sufficient data to derive them. For the systematic review, we also included studies that reported median OS or PFS comparing FGR with non-FGR. Studies without survival analyses, without direct comparisons between FGR and non-FGR, involving pediatric populations, or lacking extractable hazard ratios were excluded. Detailed inclusion and exclusion criteria are summarized in Supplementary Table S2.

### Study selection and data collection

**Fig. 1:**
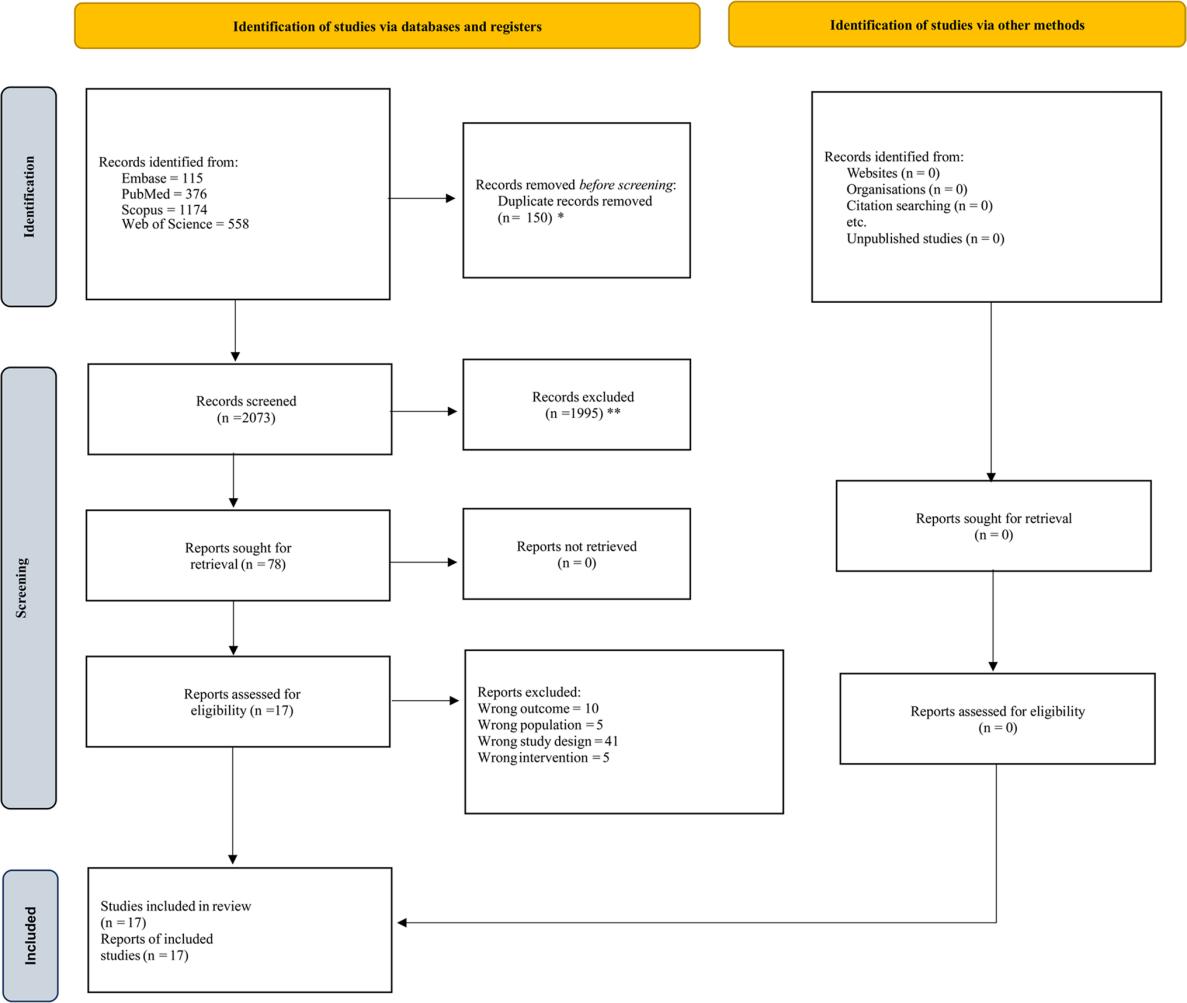
PRISMA flowchart of study selection. * With automation tool; ** Without automation tool

The process of study selection and screening was executed in strict accordance with the PRISMA 2020 guidelines. All records obtained from the database searches were imported into EndNote X9 for initial organization, where duplicate entries were systematically identified and eliminated. The curated, deduplicated dataset was subsequently transferred to Mendeley and exported in RIS format for systematic screening via the Rayyan platform (https://www.rayyan.ai/). Three independent reviewers (S.S., A.N.S., and B.D.) conducted screening of titles and abstracts, followed by a comprehensive full-text review of all studies meeting the preliminary eligibility criteria. Any discrepancies encountered during this process were resolved through consensus, with two senior investigators (A.N.S. and V.M.L.) serving as adjudicators as necessary. To ensure thoroughness, reference lists of all included studies and pertinent reviews were manually scrutinized. The entire selection process is illustrated in the PRISMA flow diagram (Fig. 1).

Eligible Studies were required to report HRs derived from multivariable Cox proportional hazards models or unadjusted HRs assessing the association between FGR versus non-FGR and OS or PFS. When available, adjusted HRs accounting for relevant clinical covariates (eg, age, extent of resection, MGMT methylation, performance status, tumor characteristics, and receipt of adjuvant therapy) were preferentially extracted to evaluate the association between fluorescence guidance and survival independent of established prognostic factors. Unadjusted HRs were extracted and analyzed separately to characterize the unadjusted associations and to assess the extent of attenuation after adjustment. For pooled analyses incorporating data from randomized controlled trials and observational cohorts, included populations were carefully cross-checked across studies to identify potential overlap. When overlap was identified, data were included only once, prioritizing the most comprehensive multivariable-adjusted analysis to avoid double-counting of patient populations.

### Risk of bias and certainty assessment

The methodological quality of the included studies was evaluated in accordance with the Cochrane Handbook for Systematic Reviews of Interventions [25]. Two independent reviewers (S.S. and A.N.S.) applied the Risk of Bias in Non-randomized Studies of Interventions (ROBINS-I) tool for the non-randomized studies [26] and the Risk of Bias tool for Randomized trials (ROB 2) tool to evaluate potential bias across domains [27]. The confidence in the evidence backing pooled estimates was evaluated using the GRADE framework, which considers risk of bias, consistency, directness, precision, and publication bias [25].

### Statistical analysis

Descriptive statistics were used to summarize the characteristics of the included studies and their patient populations. In the systematic review component, all studies demonstrating significant associations with OS and PFS in the original analyses were qualitatively synthesized. For the quantitative meta-analysis, only studies that reported time-to-event multivariable HRs or unadjusted HRs comparing FGR versus non-FGR were eligible for pooling. Unadjusted HRs were also extracted and pooled separately to characterize unadjusted associations and to assess attenuation of effect estimates following adjustment for confounding.

For each study, log hazard ratios (logHRs) and their standard errors were derived from reported HRs and 95% confidence intervals. Pooled effect estimates were obtained using a random-effects model with Hartung-Knapp correction, implemented via the meta package in R and weighted by inverse variance to address heterogeneity between studies. Statistical heterogeneity was measured with the I^2^ statistic, Cochran’s Q test, and the estimated between-study variance (τ^2^). I^2^ values > 25%, > 50%, and > 75% indicated low, considerable, and substantial heterogeneity, respectively. Separate pooled analyses were conducted for adjusted and unadjusted estimates, as well as for OS and PFS, to avoid combining both effect types. Due to expected clinical and methodological heterogeneity, including differences in study design, fluorescence modality, comparator, and covariate adjustment, pooled analyses were limited to outcomes reported by at least two comparable studies. When fewer studies were available or heterogeneity was high, results were interpreted with caution and supported by narrative synthesis. All statistical tests were two-sided, with significance set at P < 0.05. All analyses, including forest plot creation and heterogeneity evaluation, were performed using R version 4.3.2.

## Results

After an initial screening of 2,223 studies titles and abstracts, 78 full-text articles were further assessed for eligibility. After screening and eligibility assessment, a total of 17 studies, including three randomized clinical trials [1, 6, 7] and 14 observational studies, were included [8–17, 28–31]. Of these seventeen studies, thirteen reported HR [1, 6–17], along with median OS and PFS, which were used for quantitative meta-analysis; four reported median OS and PFS [28–31]. Five (29.5%) studies were conducted in Germany; two (11.8%) each in China, the United Kingdom, and France; and one (5.9%) each in the United States of America, Poland, Kazakhstan, Israel, Malaysia, and South Korea. A total of 2,849 patients were included in the analysis, comprising 1,384 in the FGR group and 1,465 in the non-FGR group. Nine studies reported unadjusted OS [1, 7, 8, 10–14, 17] for FGR compared with non-FGR, while seven reported adjusted HRs [6, 8–10, 12, 15, 16]. Three studies reported unadjusted PFS [7, 11, 13] for FGR compared with non-FGR, and four studies reported adjusted HRs [1, 6, 13, 16]. A comprehensive summary of the studies included, as well as the covariates adjusted in multivariable analyses for each study, is presented in Table 1.

**Table 1:** Summary of Included studies and their association with overall survival (OS) and progression-free survival (PFS)

Study	Country	Type of study	Period	Age group	Cancer type	Total cohort	Fluorescent group	Non-fluorescent group	OS/PFS	Covariates adjusted in the multivariable analysis	Median OS/PFS
Baig Mirza et al. [8]	UK	ROS	2013–2019	> 18 years	Glioblastoma	343	253 (5-ALA)	90 (White Light)	OS	Age, Gender, Preoperative PS, IONM, IDH status, MGMT methylation status, Radiological extent of resection, Postoperative PS, PS at 6 months Follow-up, Radiotherapy, Chemotherapy	5-ALA = 17.47 months, White light = 10.63 months
Ryskeldiyev et al. [10]	Kazakhstan	ROS	January 2022 - December 2023	> 18 years	High-grade Gliomas	141	71 (5-ALA)	70 (White Light)	OS	obesity, diabetes mellitus, ischemic heart disease, arterial hypertension, age, radiotherapy, chemotherapy, Karnofsky Performance Status (KPS), and extent of resection (EoR/GTR vs STR)	NA
Schebesch et al. [9]	Germany	POS	2013–2022	> 18 years	High-grade Gliomas	347	196 (Fluorescein sodium)	151 (White Light)	OS	Age, preoperative KPI/KPS, MGMT status, and fluorescence-guided (FY) resection	FL = 16.7 months, White light = 15.5 months
									PFS	NA	FL = 8.12 months, White light = 6.94 months
Xiao et al. [11]	China	ROS	January 2020—June 2023	> 18 years	WHO Grade III and IV	67	32 (5-ALA and Fluorescein Sodium)	35 (White Light)	OS	NA	FL = 18.2 months, White light = 14 months
									PFS	NA	FL = 11.2 months, White light = 7.7 months
Stummer et al. [1]	Germany	RCT	October 1999—July 2004	> 18 years	Malignant Glioma	270	139 (5-ALA)	131 (White Light)	OS	NA	5-ALA = 14.1 months, White light = 11.5 months
									PFS	Age, Tumor Location and KPS	5-ALA = 5.1 months, White light = 3.6 months
Ng et al. [12]	Malaysia	ROS	January 2008—December 2014	18—65 years	High-grade Gliomas	74	37 (5-ALA)	37 (White Light)	OS	Surgical method, Pre-op KPS, Histology, and Adjuvant therapy	5-ALA = 12 months, White light = 8 months
Kim et al. [29]	South Korea	ROS	January 2009—May 2011	16–81 years	Glioblastoma	80	40 (5-ALA)	40 (White Light)	OS	NA	5-ALA = 24 months, White light = 14 months
									FPS	NA	5-ALA = 18 months, White light = 6 months
Mazurek et al. [28]	Poland	ROS	May 2020 – November 2023	18—80 years	High-grade Gliomas	39	22 (5-ALA)	17 (White Light)	OS	NA	5-ALA = 9.97 months, White light = 6.4 months
Roder et al. [6]	Germany	RCT	July 2015—June 2020	18—80 years	Glioblastoma	277	127 (5-ALA)	150 (iMRI)	OS	Age, Gender, KPS, MGMT methylation status, Tumor location, Tumor volume, Extent of resection, Use of adjuvant therapy, Center/treatment allocation factors	5-ALA = 31.7 months, iMRI = 22.9 months
									PFS	Age, Gender, KPS, MGMT methylation status, Tumor location, Tumor volume, Extent of resection, Use of adjuvant therapy, Center/treatment allocation factors	5-ALA = 5.4 months, iMRI = 5.6 months
Picart et al. [13]	France	POS	November 2012—November 2015	18—80 years	Glioblastoma	51	24 (5-ALA)	27 (White Light)	OS	NA	5-ALA = 12 months, White light = 25 months
									PFS	Age, Sex, Tumor volume, Residuals KPS preoperative	5-ALA = 7 months, White light = 15 months
Wong et al. [15]	UK	ROS	2017–2020	> 18 years	High-grade Gliomas	239	50 (5-ALA)	189 (White Light)	OS	Age, Molecular status, Adjuvant therapy, Neurological status, and Extent of resection	5-ALA = 14.8 months, White light = 12.5 months
Coburger et al. [30]	Germany	ROS	September 2008—February 2014	> 18 years	Glioblastoma	177	33 (5-ALA + iMRI)	144 (iMRI)	OS	NA	5-ALA + iMRI = 18 months, iMRI = 17 months
									PFS	NA	5-ALA + iMRI = 6 months, iMRI = 6 months
Kuppler et al. [16]	Germany	ROS	2013—2023	> 18 years	Glioblastoma	128	85 (5-ALA)	43 (white Light)	OS	Age, Sex, Performance status, RPA class, MGMT methylation, Ki-67 index, Multifocality, Extent of resection, Receipt of Stupp protocol, Radiation dose, and Treatment timing variables	NA
									PFS	Age, Sex, Performance status, RPA class, MGMT methylation, Ki-67 index, Multifocality, Extent of resection, Receipt of Stupp protocol, Radiation dose, and Treatment timing variables	NA
Picart et al. [7]	France	RCT	March 2013—August 2016	> 18 years	Glioblastoma	171	88 (5-ALA)	83 (white Light)	OS	NA	5-ALA = 18.7 months, White light = 20.1 months
									PFS	NA	5-ALA = 10 months, White light = 10.3 months
Katsevman et al. [31]	USA	ROS	May 2014—June 2017	> 18 years	Glioblastoma	189	57 (Fluorescein sodium)	132 (white Light)	OS	NA	5-ALA = 18.3 months, White light = 13.96 months
Laviv et al. [14]	Israel	ROS	January 2011—December 2021	> 18 years	Glioblastoma	94	48 (5-ALA)	46 (white Light)	OS	NA	5-ALA = 15.4 months, White light = 13.76 months
									PFS	NA	5-ALA = 8.28 months, White light = 7.93 months
Zhang et al. [17]	China	ROS	2018—2024	> 18 years	Glioblastoma	162	82 (Fluorescein sodium)	80 (white Light)	OS	NA	NA

*5-ALA* 5-aminolevulinic acid, *ROS* retrospective cohort study, *POS* prospective cohort study, *RCT* randomized control study, *KPS* karnofsky performance score, *RPA* recursive partitioning analysis, *MGMT* O⁶-methylguanine–DNA methyltransferase

Within the included studies, hazard ratios for OS and PFS were derived from multivariable Cox proportional hazards models that controlled for established clinical and tumor-related prognostic factors. Although the exact covariates differed across studies, common adjustments included age, sex, baseline performance status (e.g., WHO or KPS), extent of resection, tumor grade, molecular markers (e.g., IDH mutation and MGMT promoter methylation status), adjunctive treatment, and treatment modality (see Table 1).

### Overall survival

**Fig. 2:**
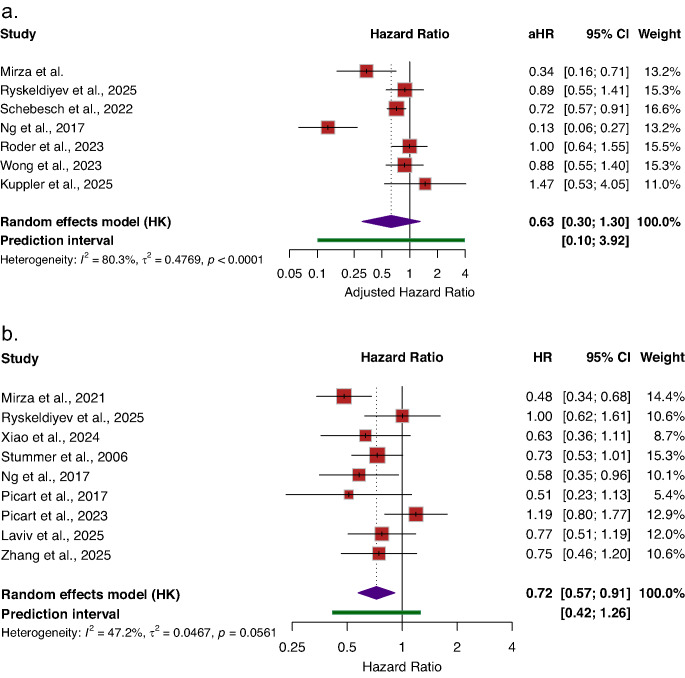
Association between Fluorescence-guided resection (FGR) and overall survival compared to Non-FGR. **a**) Adjusted overall-survival, **b**) Un-adjusted overall-survival. Reported as hazard ratios (HRs) with 95% CIs. HR < 1 indicates improved overall survival with FGR compared with Non-FGR

Nine studies reported unadjusted hazard ratios for OS comparing fluorescence-guided with non-fluorescence-guided resection. In pooled unadjusted analyses, FGR was associated with a statistically significant improvement in OS (HR = 0.72; 95% CI [0.57–0.91], *I*^*2*^ = 47.2%) compared to non-FGR (Fig. 2b). Most studies reported point estimates favoring fluorescence guidance, although confidence intervals did not exclude unity in several cohorts, reflecting variability in study populations and surgical practices.

Seven studies reported multivariable-adjusted hazard ratios for OS. These analyses accounted for key prognostic factors, including age, performance status, extent of resection, molecular characteristics, and receipt of adjuvant therapy. In contrast to the unadjusted findings, pooled adjusted analyses showed a directionally favorable but statistically nonsignificant association between FGR and OS (aHR = 0.63; 95% CI [0.30–1.30], *I*^*2*^ = 80.3%) compared with non-FGR (Fig. 2a). The diminution in the survival association after adjustment suggests that the observed survival benefits in the unadjusted analyses may be partially attributable to confounding related to patient selection and treatment heterogeneity.

### Progression free survival

**Fig. 3:**
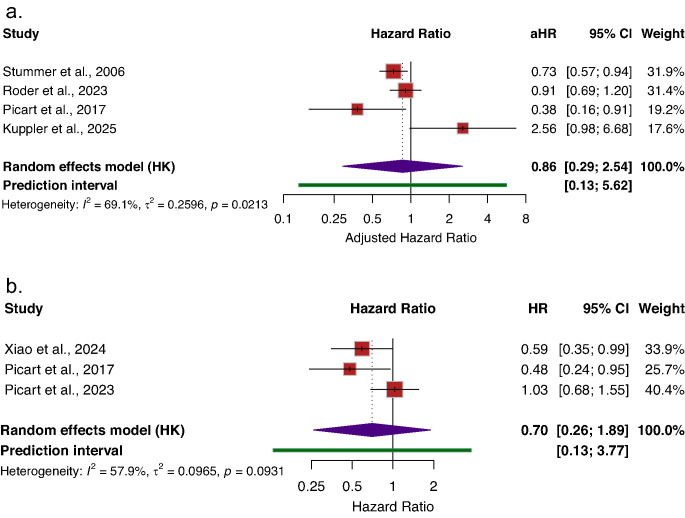
Association between Fluorescence-guided resection (FGR) and progression-free survival compared to Non-FGR. **a**) Adjusted progression-free survival, **b**) Un-adjusted progression-free survival. Reported as hazard ratios (HRs) with 95%CIs. HR < 1 indicates improved progression-free survival with FGR compared with Non-FGR

Three studies contributed unadjusted PFS estimates. In pooled analysis, FGR was associated with a non-significant trend toward improved PFS (HR = 0.70; 95% CI [0.26–1.89], *I*^*2*^ = 57.9%) compared to non-FGR (Fig. 3b). Four studies reported adjusted hazard ratios for PFS. Adjusted pooled analyses similarly demonstrated a directionally favorable but non-significant association for FGR (aHR = 0.86; 95% CI [0.29–2.54], *I*^*2*^ = 80.3%) (Fig. 3a).

### Subgroup analysis: 5-ALA versus white-light resection

**Fig. 4:**
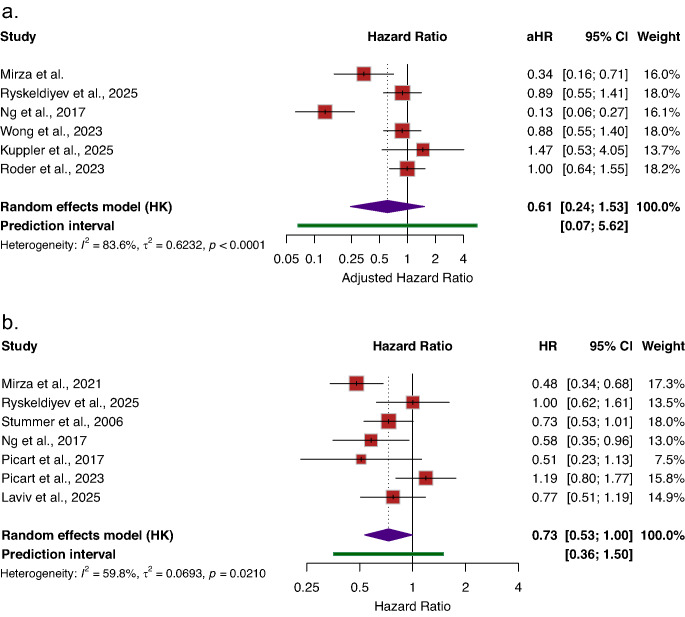
Association between 5-Aminolevulinic Acid (5-ALA) and overall survival compared to white light. **a**) Adjusted overall-survival, **b**) Un-adjusted overall-survival. Reported as hazard ratios (HRs) with 95% CIs. HR < 1 indicates improved overall survival with 5-ALA compared with white light

Subgroup analyses were conducted on studies directly comparing 5-ALA-guided resection with white-light surgery. In unadjusted analyses, 5-ALA-assisted surgery demonstrated a statistically significant improvement in OS (HR = 0.73; 95% CI [0.53–1.00], *I*^*2*^ = 59.8%) compared to white light (Fig. 4b). However, in adjusted analyses, the association was attenuated and no longer statistically significant (aHR = 0.61; 95% CI [0.24–1.53], *I*^*2*^ = 83.6%) (Fig. 4a). These observed patterns were consistent with those noted in the overall fluorescence-guided analyses.

### Risk of bias and certainty of evidence

Risk of bias assessments indicated a moderate to serious risk of bias in observational studies, primarily driven by confounding by indication, whereas randomized trials were judged to have some concerns under the RoB 2 framework due to the inability to blind surgical interventions and variability in outcome assessment (Supplementary Figs. 1 and 2).

Using the GRADE framework, the certainty of evidence supporting the association between fluorescence-guided resection and overall survival was rated as low for unadjusted analyses and very low for adjusted analyses. Downgrading was driven primarily by risk of bias related to confounding by indication in observational studies, inconsistency reflected by substantial between-study heterogeneity, and imprecision due to wide confidence intervals that frequently crossed unity in adjusted models. Indirectness was additionally considered, given variability in fluorescence modalities, comparators, and patient populations across studies. Suspected publication bias was also noted, given the predominance of single-center surgical cohorts and the limited reporting of null survival effects (Supplementary Table 3).

Certainty of evidence for progression-free survival was rated as very low in both the unadjusted and adjusted analyses, reflecting the smaller number of contributing studies, greater heterogeneity in outcome definitions and follow-up protocols, and marked imprecision of the pooled estimates. Study-level contributors to these judgments, including differences in covariate adjustment strategies, fluorescence modality, and outcome assessment, are summarized in the Supplementary Table 3.

## Discussion

In this systematic review and meta-analysis, we evaluated survival outcomes associated with fluorescence-guided versus non-fluorescence-guided resection in high-grade glioma, with explicit separation of unadjusted and adjusted time-to-event estimates. Across 13 studies included in the meta-analysis, fluorescence-guided resection was associated with significantly improved unadjusted overall survival; however, adjusted analyses showed attenuation of effect and loss of statistical significance for both OS and PFS. These findings indicate that, although fluorescence-guided surgery is consistently associated with favorable survival outcomes in observational comparisons, the current evidence does not support a definitive independent survival benefit after adjusting for confounding.

The distinction between technical efficacy and survival causality is well supported by existing literature [4, 32, 33]. Fluorescence-guided surgery was developed to enhance intraoperative tumor visualization and increase the extent of resection, a surgical endpoint repeatedly associated with improved oncologic outcomes in glioblastoma [1, 18, 32]. The landmark multicenter randomized controlled trial by Stummer et al. demonstrated significantly higher rates of complete resection of contrast-enhancing tumors and improved PFS with 5-ALA-guided surgery, whereas no statistically significant difference in OS was observed between treatment arms [1]. Subsequent analyses and observational cohorts have reinforced that fluorescence guidance reliably improves resection completeness, supporting its role as a technical adjunct rather than a direct modifier of tumor biology [8–17]. Our findings align with this paradigm, with directionally favorable but imprecise PFS estimates and attenuated OS effects after adjustment.

The attenuation of survival associations in adjusted analyses likely reflects confounding by indication and treatment heterogeneity, which are pervasive challenges in surgical oncology research. Patients selected for fluorescence-guided resection often differ systematically from those undergoing conventional surgery, including better baseline performance status, more favorable tumor locations, higher likelihood of achieving gross total resection, treatment at high-volume centers, and greater access to contemporary adjuvant therapy [7, 10, 34–36]. Numerous studies have demonstrated that the extent of resection, rather than the surgical adjunct itself, is the factor most consistently associated with survival in high-grade glioma [18, 32, 37]. Even after multivariable adjustment, residual confounding from unmeasured factors such as surgeon expertise, institutional protocols, molecular subclassification, and postoperative treatment timing may persist, limiting causal inference [38–41].

Importantly, inconsistent survival findings across studies should not be interpreted as evidence against the clinical value of fluorescence-guided surgery [1, 32, 42, 43]. Instead, they underscore the complexity of linking intraoperative technologies to long-term outcomes in biologically aggressive tumors [1, 32, 42]. FGR operates proximally in the care pathway by improving cytoreduction, whereas OS is heavily influenced by downstream factors, including tumor molecular profile, responsiveness to chemoradiation, and patterns of recurrence [1, 20, 34, 44]. The directionally favorable trends observed for PFS in our analysis are biologically plausible and concordant with prior randomized evidence demonstrating improved local disease control with fluorescence guidance [1, 20, 45]. Variability in PFS definitions, imaging intervals, and assessment criteria across studies likely contributed to the imprecision observed in pooled PFS estimates [46–49].

Subgroup analyses of 5-ALA versus white-light resection showed patterns consistent with the overall analysis, with borderline improvements in unadjusted OS that were attenuated after adjustment. These findings mirror prior reports in which survival advantages associated with 5-ALA use diminish after controlling for key prognostic factors [4, 6]. Importantly, our analysis was not designed to compare fluorescence agents directly, and differences between 5-ALA and fluorescein sodium should not be inferred. Rather, the consistent attenuation across fluorescence modalities reinforces the interpretation that survival signals are influenced by confounding and study design rather than fluorescence agent-specific effects.

### Strengths and limitations

Our study addresses key limitations of prior meta-analyses, which have often pooled heterogeneous study designs and relied predominantly on unadjusted survival estimates. By explicitly separating unadjusted and adjusted hazard ratios, our analysis clarifies how survival associations attributed to fluorescence-guided surgery are sensitive to adjustment and heterogeneity. This approach provides a more nuanced and methodologically robust interpretation of the existing literature and highlights the importance of analytic rigor when evaluating surgical adjuncts.

Several limitations warrant consideration. Most included studies were observational, and adjusted analyses remain susceptible to residual confounding. Substantial heterogeneity was observed, reflecting variability in fluorescence modality, comparator, covariate adjustment strategies, and treatment era. Importantly, adjusted survival estimates were derived from multivariable models incorporating differing sets of prognostic variables across studies. As such, pooled adjusted effects may not represent strictly comparable exposure contrasts but rather study-specific estimands conditional on distinct confounding structures. This heterogeneity in adjustment strategies may contribute to residual bias and should be considered when interpreting pooled adjusted estimates. PFS was inconsistently reported and variably defined, limiting precision and certainty of evidence. Finally, publication bias cannot be excluded, particularly given the predominance of single-center cohorts and selective reporting of favorable outcomes.

An additional consideration is the potential for treatment crossover during repeat surgeries. In cases of glioblastoma, salvage resections are frequently performed, and the utilization of fluorescence during subsequent procedures may vary from that used in the initial operation. Since overall survival is influenced by the cumulative therapeutic interventions, variability in fluorescence application at recurrence could confound the independent effect of the primary approach. Most of the included studies did not systematically report crossover rates, thereby limiting the capacity to adjust for this factor. Future prospective trials should consistently document fluorescence usage across all surgical interventions to more accurately determine its impact on survival outcomes.

## Conclusions

In summary, fluorescence-guided resection is consistently associated with improved unadjusted survival outcomes in high-grade glioma; however, adjusted analyses do not demonstrate a definitive independent survival benefit. These findings support fluorescence guidance as a valuable intraoperative adjunct that enhances surgical visualization and facilitates maximal safe resection, rather than as a survival-modifying intervention in isolation. Future randomized trials incorporating contemporary molecular stratification, standardized outcome assessment, and modern adjuvant therapy will be essential to clarify the extent to which improved surgical guidance translates into durable survival benefits.

## Supplementary Information

Below is the link to the electronic supplementary material.

Supplementary file1 (DOCX 384 KB)

## Data Availability

The datasets generated during and/or analysed during the current study are available from the corresponding author on reasonable request.
