# Supplementary material for: Fluorescence-guided versus non-fluorescence-guided resection in high-grade glioma: a systematic review and meta-analysis of survival outcomes
Source: Neurosurg Rev. 2026 Apr 23;49(1):370. doi: 10.1007/s10143-026-04297-8 (PMC13102904; doi:10.1007/s10143-026-04297-8)
Supplement: Supplementary file 1 — Supplementary file1 (DOCX 384 KB) [file 10143_2026_4297_MOESM1_ESM.docx]

**Supplementary Table 1.** Search Strategy

| PUBMED/MEDLINE | (Glioblastoma[tiab] OR glioblastoma*[tiab] OR GBM [tiab]  OR ("high grade"[tiab] AND glioma*[tiab]) OR HGG [tiab])  AND  ("5-aminolevulinic acid"[tiab] OR 5-ALA [tiab] OR aminolevulinic[tiab]  OR fluorescein[tiab] OR "fluorescein sodium"[tiab]  OR fluorescen*[tiab] OR "fluorescence-guided"[tiab] OR "fluorescence guided"[tiab])  AND  (resect*[tiab] OR surg*[tiab] OR craniotom*[tiab]  OR "tumor resection"[tiab] OR "extent of resection"[tiab]  OR gross-total[tiab] OR "gross total"[tiab] OR GTR [tiab])  AND  (random*[tiab] OR trial[tiab] OR cohort[tiab] OR comparative[tiab]  OR comparison[tiab] OR matched[tiab] OR propensity[tiab]  OR control*[tiab] OR versus[tiab] OR vs[tiab])  NOT  (pediatric[tiab] OR paediatric[tiab] OR child*[tiab] OR adolescent*[tiab]) | 376 |
| --- | --- | --- |
| EMBASE | (glioblastoma OR GBM OR "high grade glioma" OR HGG)  AND  ("5-aminolevulinic acid" OR "5-ALA" OR aminolevulinic  OR fluorescein OR "fluorescein sodium" OR "fluorescence-guided" OR "fluorescence guided")  AND  (resection OR surgery OR craniotomy OR "extent of resection" OR GTR OR "gross total")  AND  (random* OR trial OR cohort OR comparative OR comparison  OR matched OR propensity OR control* OR versus OR vs)  NOT  (pediatric OR paediatric OR child OR adolescent) | 115 |
| SCOPUS | TITLE-ABS-KEY (glioblastoma* OR GBM OR ("high grade" W/2 glioma*) OR HGG)  AND  TITLE-ABS-KEY ("5-aminolevulinic acid" OR "5-ALA" OR aminolevulinic OR fluorescein OR "fluorescein sodium"OR fluorescen* OR "fluorescence-guided" OR "fluorescence guided")  AND  TITLE-ABS-KEY (resect* OR surg* OR craniotom*OR "tumor resection" OR "extent of resection"  OR GTR OR "gross total")  AND  TITLE-ABS-KEY (random* OR trial OR cohort OR comparative OR comparison OR matched OR propensity OR control* OR versus OR vs)  AND  NOT TITLE-ABS-KEY(pediatric OR paediatric OR child* OR adolescent*) | 1174 |
| WEB OF SCIENCE | (glioblastoma OR GBM OR "high grade glioma" OR HGG)  AND  ("5-aminolevulinic acid" OR 5-ALA OR fluorescein OR "fluorescein sodium" OR fluorescence-guided OR "fluorescence guided")  AND  (resection OR surgery OR craniotomy OR "extent of resection" OR GTR OR "gross total")  AND  (random* OR trial OR cohort OR comparative OR comparison OR matched OR propensity OR control* OR versus OR vs)  NOT  (pediatric OR paediatric OR child OR adolescent) | 558 |

**Supplementary Table 2.** Inclusion and exclusion criteria

| **Inclusion Criteria** | **Exclusion Criteria** |
| --- | --- |
| - Randomized controlled trials (RCTs) and comparative observational cohort studies (prospective or retrospective) - Adult patients (≥18 years) - High-grade glioma, including WHO grade III–IV gliomas or glioblastoma - Fluorescence-guided resection using 5-aminolevulinic acid (5-ALA), fluorescein sodium, or mixed fluorescence modalities - Non-fluorescence-guided resection (white-light microscopy or other non-fluorescence techniques, including iMRI when used as a comparator) - Overall survival (OS) and/or progression-free survival (PFS) reported as time-to-event outcomes - Hazard ratios (HRs) with 95% confidence intervals derived from unadjusted analyses or multivariable Cox proportional hazards models - Studies reporting multivariable-adjusted HRs accounting for relevant prognostic covariates (e.g., age, performance status, extent of resection, molecular markers, adjuvant therapy) or unadjusted HRs (analyzed separately) - Full-text articles published in English - When overlapping cohorts were identified, only the most comprehensive or methodologically rigorous analysis was included - Comparative cohorts with ≥10 patients per treatment arm | - Case reports, case series, editorials, narrative reviews, systematic reviews, conference abstracts without full text - Pediatric populations (<18 years) - Low-grade gliomas, metastatic brain tumors, non-glial intracranial tumors - Studies without intraoperative fluorescence guidance - Non-comparative studies or studies lacking a clearly defined non-fluorescence control group - Studies reporting only extent of resection, complication rates, or functional outcomes without survival data - Studies reporting survival outcomes without HRs or insufficient data to derive HRs - Studies pooling adjusted and unadjusted estimates without distinction - Non-English publications without accessible translations - Duplicate publications reporting overlapping patient populations without additional data - Very small cohorts (<10 patients per arm) |

**Supplementary Table 3.** Certainty of evidence of the included studies

| **Outcome** | **Risk of Bias** | **Inconsistency** | **Indirectness** | **Imprecision** | **Publication Bias** | **Overall Certainty** |
| --- | --- | --- | --- | --- | --- | --- |
| OS (unadjusted) | Serious | Serious | Serious | Not serious | Suspected | LOW ⨁⨁◯◯ |
| OS (adjusted) | Serious | Very serious | Serious | Serious | Suspected | VERY LOW ⨁◯◯◯ |
| PFS (unadjusted) | Very serious | Serious | Serious | Very serious | Suspected | VERY LOW ⨁◯◯◯ |
| PFS (adjusted) | Serious | Very serious | Serious | Very serious | Suspected | VERY LOW ⨁◯◯◯ |
| OS: 5-ALA vs WL (unadjusted) | Serious | Serious | Serious | Serious | Suspected | LOW ⨁⨁◯◯ |
| OS: 5-ALA vs WL (adjusted) | Serious | Very serious | Serious | Serious | Suspected | VERY LOW ⨁◯◯◯ |

**Supplementary Figure 1.** Risk of Bias Assessment using ROBINS-I tool

**Supplementary Figure 2.** Risk of Bias Assessment using Risk of Bias tool for Randomized trials (ROB-II).
